# Paired Urinary Cytology and Targeted FGFR3 S249C/R248C Mutation Detection in Urine from Patients with Bladder Cancer: A Pilot Study

**DOI:** 10.3390/jcm15187117

**Published:** 2026-09-14

**Authors:** Bogdan-Petru Tichil, Anamaria Besleaga, Ioana Gheorghiu, Alin-Dan Chiorean, Mihaela Laura Vica Matei, Adrian Florea

**Affiliations:** 1 Department of Cell and Molecular Biology, Faculty of Medicine, University of Medicine and Pharmacy “Iuliu Hatieganu”, 400349 Cluj-Napoca, Romania; tichil.bogdan.petru@elearn.umfcluj.ro (B.-P.T.); chiorean_alin_dan@yahoo.com (A.-D.C.); adrian_a_florea@yahoo.com (A.F.); 2 Deva Emergency County Hospital, 330084 Deva, Romania; besleaga.anamaria@elearn.umfcluj.ro or secretariat@spital-deva.ro; 3 Department of Otorhinolaryngology, University of Medicine and Pharmacy “Iuliu Hatieganu”, 400349 Cluj-Napoca, Romania; 4 Department of Medical Informatics and Biostatistics, University of Medicine and Pharmacy “Iuliu Hatieganu”, 400349 Cluj-Napoca, Romania; taranu.ioana@umfcluj.ro; 5 Clinical Institute of Urology and Renal Transplant, 400000 Cluj-Napoca, Romania

**Keywords:** bladder cancer, urothelial carcinoma, FGFR3, S249C, R248C, urinary cytology, urinary biomarkers, real-time PCR, non-muscle-invasive bladder cancer

## Abstract

**Background**: Urinary cytology is routinely used as an adjunct to cystoscopy in the diagnosis and surveillance of urothelial carcinoma. Although it has high specificity for high-grade tumors, its sensitivity for low-grade papillary lesions is limited. Activating mutations in the fibroblast growth factor receptor 3 (FGFR3) gene are frequently encountered in non-muscle-invasive bladder cancer and may provide a complementary molecular method for detecting tumors that are not identified by conventional cytology. **Objective**: To compare conventional urinary cytology with urinary detection of the FGFR3 hotspot mutations S249C and R248C in patients undergoing evaluation for urothelial carcinoma. **Materials and Methods**: This single-center prospective pilot study included adult patients with clinically and radiologically confirmed bladder tumors evaluated between February 2022 and December 2023. Voided urine specimens were collected before cystoscopy or urinary tract instrumentation. Urine cytology was performed after centrifugation, conventional smear preparation, alcohol fixation, and Papanicolaou staining, with interpretation according to The Paris System for Reporting Urinary Cytology. DNA extracted from paired urine specimens was analyzed for the FGFR3 S249C and R248C hotspot mutations using TaqMan mutation-detection assays and real-time polymerase chain reaction. Histopathological examination of transurethral resection, bladder biopsy, or radical surgical specimens served as the reference standard. **Results**: Twenty-six patients were included, with a mean age of 69.2 ± 11.5 years; 24 patients (92.3%) were male. At least one FGFR3 mutation was detected in 17 patients (65.4%), whereas nine patients (34.6%) had no detectable mutation. The S249C mutation was identified in three patients, while the R248C mutation was detected in nine patients, 5 patients were positive for both mutations.

## 1. Introduction

Bladder cancer (BC) is among the ten most frequently diagnosed malignancies worldwide and remains a major cause of morbidity, mortality, and healthcare expenditure. More than 600,000 new cases and 220,000 deaths occur annually, with increasing incidence because of population aging and continued exposure to carcinogens such as tobacco smoke and occupational aromatic amines [1]. More than 90% of bladder tumors are urothelial carcinomas; approximately 70–75% present as non-muscle-invasive bladder cancer (NMIBC), including Ta, T1, and carcinoma in situ (CIS), while the remainder are muscle-invasive at diagnosis [2,3,4]. Although NMIBC generally has favorable cancer-specific survival, 50–70% of patients experience recurrence and approximately 10–20% progress to muscle-invasive disease [5,6]. Repeated endoscopic procedures and prolonged surveillance consequently make bladder cancer one of the most expensive malignancies to manage per patient [7].

Guidelines from the European Association of Urology (EAU), American Urological Association/Society of Urologic Oncology (AUA/SUO), and National Comprehensive Cancer Network (NCCN) identify cystoscopy with histopathological confirmation following transurethral resection as the diagnostic standard [2,3,4]. Cystoscopy permits direct lesion visualization and tissue acquisition, while NMIBC surveillance remains based mainly on periodic cystoscopy because of the high recurrence risk [2,3]. Urinary biomarkers may provide complementary information in selected situations, but current molecular assays cannot replace cystoscopy across the full spectrum of urothelial carcinoma [2,3,4]. Urinary cytology has been used routinely for decades and remains recommended as an adjunct, particularly for suspected high-grade urothelial carcinoma, CIS, unexplained positive urinary findings despite negative cystoscopy, and surveillance of intermediate- or high-risk NMIBC [2,3,4]. Its principal value lies in detecting high-grade disease and providing cellular information that complements endoscopic findings [2,3,4].

Urine cytology is based on microscopic examination of exfoliated urothelial cells obtained from voided urine, bladder washings, or instrumented specimens. Malignant cells may show nuclear enlargement, hyperchromasia, coarse chromatin, irregular contours, increased nuclear-to-cytoplasmic ratio, and prominent nucleoli [8]. High-grade tumors generally exfoliate abundant atypical cells, whereas low-grade papillary tumors often retain near-normal architecture and release few abnormal cells; performance therefore depends on tumor biology, specimen quality, and cytopathological expertise [9,10]. Cytology has very high specificity, usually above 90% and often approaching 95–100% in experienced laboratories [11,12,13]. Overall sensitivity is lower, commonly 35–55%, and varies markedly by grade [11,14]. Sensitivity for high-grade carcinoma and CIS often reaches 70–90% or more [9,11,15], whereas low-grade tumors are frequently detected in fewer than 30% of cases because their cytological atypia is limited [11,14].

These limitations prompted the development of The Paris System for Reporting Urinary Cytology (TPS), which focuses primarily on identifying high-grade urothelial carcinoma rather than attempting reliable diagnosis of all urothelial neoplasms [16,17,18]. Its standardized categories—Negative for High-Grade Urothelial Carcinoma, Atypical Urothelial Cells, Suspicious for High-Grade Urothelial Carcinoma, and High-Grade Urothelial Carcinoma—improve communication and reduce interobserver variability [16,17,18,19]. Validation studies indicate that TPS enhances reproducibility without sacrificing specificity [17,18,19,20]. Nevertheless, cytological interpretation remains influenced by cellularity, fixation, preparation, and observer experience. Infection, instrumentation, intravesical Bacillus Calmette–Guérin therapy, urolithiasis, radiation, and inflammation may produce reactive atypia [8,16], while hypocellular specimens or poorly exfoliating tumors can cause false-negative results. Cytology therefore has strong positive predictive value but modest negative predictive value, especially for low-grade disease [11,12,13,14,15]. It is relatively inexpensive, generally costing US$30–100 per specimen [21,22], although repeated testing, confirmatory cystoscopy, and dependence on specialist interpretation limit its economic and diagnostic impact [17,18].

The poor sensitivity of cytology for low-grade tumors has encouraged the development of molecular urinary biomarkers that detect tumor-associated alterations independently of cell morphology. Among these, activating fibroblast growth factor receptor 3 (FGFR3) mutations are especially relevant because they arise early and are common in low-grade papillary NMIBC. FGFR3 encodes a transmembrane receptor tyrosine kinase involved in proliferation, differentiation, migration, apoptosis, and tissue homeostasis through pathways including RAS–MAPK, PI3K–AKT, PLCγ, and STAT [5,23,24]. Activating point mutations cause ligand-independent receptor dimerization and constitutive signaling, promoting urothelial tumorigenesis [24,25,26].

More than ten activating FGFR3 mutations have been described, but R248C, S249C, G372C, and Y375C account for most cases [24,27]. S249C is the most frequent mutation, followed by R248C, and together they represent more than half of FGFR3-mutated urothelial tumors [24]. Mutation frequencies may approach 70–80% in low-grade Ta NMIBC but are substantially lower in high-grade and muscle-invasive disease [23,25,28]. This distribution complements cytology: cytology performs best in high-grade tumors, whereas FGFR3 alterations predominate in low-grade lesions that often escape morphological detection. FGFR3-mutated low-grade tumors generally show lower proliferative and metastatic potential and a more indolent course than tumors driven by TP53 or RB1 alterations [29]. Although recurrence remains frequent, progression is comparatively uncommon, supporting FGFR3 activation as a distinct pathway of urothelial carcinogenesis and a potential surveillance marker [24,29].

Urinary FGFR3 assays detect tumor-derived DNA released into urine and may remain informative when intact atypical cells are scarce. Available platforms include allele-specific, real-time, and multiplex polymerase chain reaction, droplet digital polymerase chain reaction, and next-generation sequencing [30,31,32]. Modern assays can detect low mutant-allele fractions within abundant wild-type DNA [31]. Across studies, specificity has generally been high, although the performance of individual assays varies according to mutation prevalence, disease characteristics, specimen type, and analytical method [30,33]. Zuiverloon and colleagues demonstrated the feasibility of FGFR3 mutation analysis in voided urine during surveillance of patients with low-grade non-muscle-invasive bladder cancer [33], while other studies have similarly supported the potential role of urinary molecular testing in recurrence surveillance [34,35].

The present study therefore compared conventional urinary cytology with targeted urinary FGFR3 hotspot analysis for S249C and R248C in paired urine specimens from patients with histopathologically confirmed urothelial carcinoma. Paired test patterns and discordance were assessed descriptively and examined in relation to clinicopathological characteristics. The study was designed as a pilot, hypothesis-generating analysis of whether these two urine-based approaches provide non-identical and potentially complementary information rather than as a formal diagnostic-accuracy study.

## 2. Materials and Methods

### 2.1. Study Design and Patients

We conducted a single-center observational pilot study with prospective data collection between February 2022 and December 2023 at the Clinical Institute of Urology and Renal Transplantation, Cluj-Napoca, Romania. Eligible patients were adults with diagnosed via cystoscopy or imaging techniques with bladder tumors. Upon enrollment in the pilot study, patients did not have pathological reports regarding tumor stage or grade. Exclusion criteria were age < 18 years, pregnancy, active urinary tract infection, another confirmed or suspected malignancy, previous radical cystectomy, or previous intravesical Bacillus Calmette–Guérin or intravesical chemotherapy. The final analysis included 26 patients with clinical and radiological confirmation of bladder tumors in whom urothelial carcinoma was confirmed. Histopathology was used to characterize the tumors; because all patients in the analyzed cohort had urothelial carcinoma, it could not provide a disease-negative comparison group for formal diagnostic-accuracy calculations.

### 2.2. Urine Collection and Cytology

Freshly voided urine was collected before TurB/bladder biopsy. When possible, first morning urine was avoided to reduce cellular degeneration associated with prolonged intravesical retention. The whole urine sample was divided into aliquots for cytological and molecular analyses. Cytology specimens were transported promptly to the Department of Pathology and processed within approximately 2–4 h. Whole urine samples were centrifuged at 600× *g* for 10 min; the supernatant was discarded and the cellular pellet was used for conventional smear preparation. Slides were fixed in an alcohol-based fixative and stained using the Papanicolaou method; May–Grünwald–Giemsa staining was used when required for morphological assessment.

Cytological specimens were interpreted according to TPS, Second Edition, using the categories Negative for High-Grade Urothelial Carcinoma (NHGUC), Atypical Urothelial Cells (AUC), Suspicious for High-Grade Urothelial Carcinoma (SHGUC), High-Grade Urothelial Carcinoma (HGUC), Low-Grade Urothelial Neoplasm, or Other Malignancies. For the descriptive paired analysis, SHGUC and HGUC were categorized as positive cytology; all remaining results are referred to as non-positive rather than as true-negative results. Cytological assessment was performed without knowledge of the urinary FGFR3 result.

### 2.3. Urine-Derived DNA Preparation and FGFR3 Mutation Testing

DNA extraction from midstream urine was performed with the QIAamp DNA Mini Kit (Manufacturer: Qiagen, City: Hilden, Country: Germany), according to the manufacturer’s instructions. DNA obtained from the urine aliquot was assessed using a Pearl^®^ NanoPhotometer (Manufacturer: Implen GmbH, City: Munich, Country: Germany) by ultraviolet absorbance and the A260/A280 ratio. Samples with an A260/A280 ratio < 1.7 underwent additional purification with the MasterPure™ Complete DNA and RNA Purification Kit (Manufacturer: Biosearch Technologies, City: Hoddesdon, Country: UK). Purification included protein precipitation with MPC reagent, centrifugation at ≥10,000× *g*, isopropanol precipitation, two washes with 75% ethanol, and resuspension of the nucleic-acid pellet in 35 µL nuclease-free water.

Targeted mutation detection was performed using TaqMan^®^ Mutation Detection Assays with castPCR™ technology (Manufacturer: Thermo Fisher Scientific, City: Waltham, State: MA, Country: USA). The FGFR3 gene-reference assay was FGFR3_rf (Assay ID Hs00001015_rf). The R248C hotspot was tested with FGFR3_714_mu (Assay ID Hs00000811_mu), and the S249C hotspot with FGFR3_715_mu (Assay ID Hs00000812_mu). DNA was adjusted to approximately 10 ng/µL. Each 20 µL reaction contained 10 µL TaqMan Genotyping Master Mix (2×), 2 µL genomic DNA (20 ng), 2 µL of the corresponding TaqMan assay, and 6 µL nuclease-free water. A nuclease-free-water no-template control was included in each analytical run. Positive mutation controls were not used because they were not available during the study.

Amplification was performed on a QuantStudio™ 5 Real-Time PCR System (Manufacturer: Thermo Fisher Scientific, City: Waltham, State: MA, Country: USA) in standard run mode. Thermal cycling consisted of 95 °C for 10 min; five cycles of 92 °C for 15 s and 58 °C for 1 min; and 40 cycles of 92 °C for 15 s and 60 °C for 1 min, with fluorescence acquisition in the FAM channel. Real-time PCR data were analyzed with a manual fluorescence threshold of 0.2 and automatic baseline correction. For each mutation target, the mutant assay was interpreted relative to the paired FGFR3 reference assay. ΔCt was calculated as Ct (mutant assay) − Ct (reference assay), and mutation calling was based on whether the resulting ΔCt met the assay-specific detection cutoff used for that assay. The assay information available for the study did not specify a separate numerical analytical limit of detection; therefore, no independent limit-of-detection value is claimed. Reference-assay amplification curves were reviewed to confirm the presence of adequate amplifiable DNA before interpreting the mutation assay.

Each mutant and reference assay was initially performed as a single technical replicate per sample. Samples generating an invalid, inconclusive, or technically uninterpretable result were retested with the same assay from the original DNA extract according to the manufacturer’s instructions, and the repeat result was used for final interpretation. Molecular assessment was performed blinded to both urinary cytology and histopathological findings, and molecular results were recorded before integration with the clinicopathological dataset.

### 2.4. Histopathological Evaluation

Tissue obtained by transurethral resection, bladder biopsy, or radical surgery was evaluated by genitourinary pathologists according to the 2022 World Health Organization Classification of Urinary and Male Genital Tumours. Pathological stage was assigned using the TNM classification and tumor grade was classified as low-grade or high-grade urothelial carcinoma where applicable.

### 2.5. Statistical Analysis

Given the pilot nature of the study and the sample size of 26 patients, the analysis is descriptive. Categorical variables are presented as counts and percentages and age as mean ± standard deviation. Paired cytology and FGFR3 results are shown in cross-tabulated form. No inferential hypothesis testing was performed. Sensitivity, specificity, positive predictive value, negative predictive value, and overall diagnostic accuracy were not calculated because the analyzed cohort did not include a disease-negative control group.

## 3. Results

### 3.1. Cohort and Tumor Characteristics

Twenty-six patients were included. Mean age was 69.2 ± 11.5 years and 24/26 patients (92.3%) were male. Histopathological stage was Ta in 16 patients (61.5%), T1 in 3 (11.5%), and T2 in 7 (26.9%). The principal clinicopathological and urinary test findings are summarized in Table 1.

### 3.2. Paired Urinary Cytology and FGFR3 Results

At least one targeted FGFR3 mutation was detected in 17/26 patients (65.4%), whereas cytology was positive in 8/26 patients (30.8%). Five patients were positive by both approaches. Twelve patients had one or both targeted FGFR3 mutations despite non-positive cytology, while three had positive cytology without detectable S249C or R248C. Six patients had neither positive cytology nor either targeted mutation. Thus, the two urine-based approaches were discordant in 15/26 patients (57.7%) (Table 2). This discordance is reported descriptively and is not interpreted as evidence of superiority of either method.

**Figure 1:**
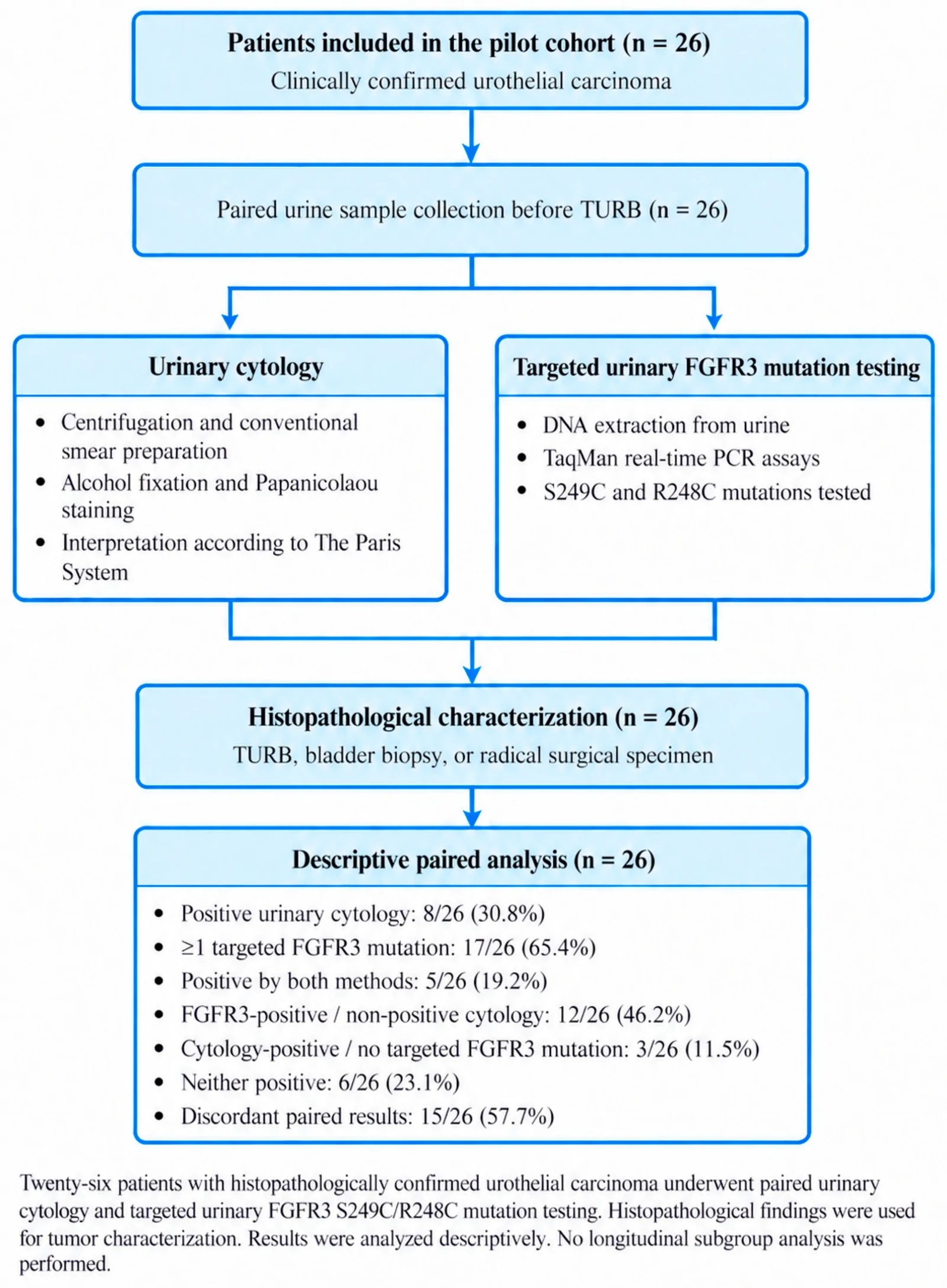
Patient flow and paired urinary testing in the pilot cohort.

Within the Ta subgroup, 11/16 patients (68.8%) had at least one targeted FGFR3 mutation and 4/16 (25.0%) had positive cytology. Among the combined T1/T2 subgroup, 6/10 patients (60.0%) had at least one targeted mutation and 4/10 (40.0%) had positive cytology. These subgroup counts are descriptive only and were not subjected to significance testing Figure 1.

## 4. Discussion

This pilot study was focused on a single question: how conventional urinary cytology and targeted urinary detection of FGFR3 S249C/R248C behave when assessed in the same small cohort. The main finding is the frequency of discordant paired results. Although 17/26 patients had at least one targeted FGFR3 mutation, only 8/26 had positive cytology; 12 patients were FGFR3-positive despite non-positive cytology, while 3 showed the opposite pattern. These observations do not demonstrate that FGFR3 testing is more sensitive than cytology because the study lacks disease-negative controls, comprehensive tumor-tissue genotyping, and adequate sample size for a formal diagnostic-accuracy analysis. Rather, they support the narrower hypothesis that morphology-based cytology and mutation-specific urinary testing can provide non-identical information.

The biological rationale for such discordance is plausible. FGFR3 mutations are particularly frequent in papillary NMIBC, and S249C and R248C are among the recurrent activating hotspots described in urothelial carcinoma [23,24,25,26,28]. By contrast, urinary cytology is primarily optimized for recognition of high-grade morphological atypia and performs substantially less well in many low-grade papillary tumors [8,9,11,12,13,14,15]. Rieger-Christ et al. demonstrated that FGFR3 mutations detected in urine sediment DNA could complement cytology in bladder tumor detection [27]. Subsequent studies further demonstrated the feasibility of urinary FGFR3 mutation testing in NMIBC surveillance [29,30]. The findings of the present pilot study are compatible with this concept but do not allow quantification of incremental diagnostic benefit. The study by Bannier et al. provides an important contemporary context for FGFR3 testing [36]. Using 1222 cases, the investigators developed and externally validated a deep-learning model that pre-screened FGFR3 mutational status from routine H&E-stained tumor slides. The model achieved > 93% sensitivity in advanced and metastatic validation cohorts while potentially reducing molecular testing by approximately 40%. Notably, S249C and R248C were among the recurrent hotspots represented in their dataset, and S249C was reported as the easiest hotspot for the model to detect. Their approach is fundamentally different from ours: Bannier et al. inferred mutation probability from tumor histomorphology and used tissue molecular status as ground truth, whereas the present study directly interrogated two predefined mutations in urine-derived DNA and did not perform paired tumor-tissue FGFR3 genotyping [36]. The comparison therefore highlights both the promise of alternative FGFR3 pre-screening strategies and the stronger validation framework required before clinical implementation.

The 2026 ASCO Genitourinary Cancers Symposium report by Basin et al. further illustrates the importance of biospecimen type and tumor-derived DNA fraction in molecular FGFR3 testing [37]. In clinically advanced urothelial bladder cancer, the investigators compared large tissue-biopsy and plasma liquid-biopsy datasets and found that detection of FGFR3 alterations in circulating tumor DNA increased with plasma tumor fraction; when tumor fraction was <1%, FGFR3-positive disease could be missed. These results concern plasma circulating tumor DNA, not urine, and should not be extrapolated directly to urinary assays. They nevertheless reinforce a relevant principle for the present work: the analytical yield of a non-tissue molecular assay depends on the biological source and quantity of tumor-derived DNA, and negative results cannot be interpreted independently of these factors.

The clinical relevance of urinary FGFR3 testing in this setting is therefore complementary rather than substitutive. Cystoscopy and histopathology remain essential for diagnosis and risk stratification, and cytology remains clinically valuable for high-grade disease [1,2,3,4,5,6,7]. A targeted urinary assay that interrogates only S249C and R248C cannot detect FGFR3-wild-type tumors or tumors carrying other FGFR3 alterations. Conversely, mutation detection in urine does not by itself establish the presence, location, grade, or stage of a tumor. The potential value of combining cytology with molecular urine testing must therefore be demonstrated prospectively using predefined clinical endpoints rather than inferred from mutation prevalence alone.

Several limitations are central to interpretation of this pilot study. First, the cohort comprised only 26 patients from a single center, making inferential statistical testing inappropriate. Second, all analyzed patients had histopathological confirmed urothelial carcinoma; without a disease-negative control group, sensitivity, specificity, predictive values, and diagnostic accuracy cannot be estimated. Third, only S249C and R248C were tested, so a negative urinary result does not indicate FGFR3 wild-type status. Fourth, tumor tissue was not genotyped for FGFR3 in parallel; therefore, concordance between urinary mutations and the molecular status of the corresponding tumor cannot be determined. Fifth, the study did not quantify mutant allele fraction or evaluate broader genomic panels.

The principal strength is the prospective paired assessment of cytology and targeted FGFR3 testing from the same urine collection in a clinically characterized cohort. This design permits a direct description of within-patient discordance without implying that either test is a diagnostic reference standard. The findings can therefore serve as pilot data for a larger study that includes disease-negative controls, comprehensive tissue and urine FGFR3 profiling, prespecified cytology categories, and clinically meaningful endpoints such as detection of recurrence or high-grade disease.

## 5. Conclusions

In this 26-patient pilot study, conventional urinary cytology and targeted urinary detection of FGFR3 S249C/R248C frequently yielded discordant paired results. This pattern is compatible with the two methods interrogating different biological features of urothelial carcinoma, but it does not establish diagnostic superiority, sensitivity, or clinical utility. Targeted urinary FGFR3 testing should not replace cystoscopy, histopathology, or cytology based on these data. Larger prospective studies should include disease-negative controls, paired tumor-tissue genotyping, broader FGFR3 and genomic panels, and predefined clinical endpoints to determine whether urinary molecular testing adds clinically meaningful information to conventional evaluation.

## Figures and Tables

**Table 1:** Principal characteristics and urinary test results of the pilot cohort (n = 26).

Characteristic	Value
Age, years, mean ± SD	69.2 ± 11.5
Male sex	24 (92.3%)
Pathological stage Ta	16 (61.5%)
Pathological stage T1	3 (11.5%)
Pathological stage T2	7 (26.9%)
Positive urinary cytology	8 (30.8%)
At least one targeted FGFR3 mutation	17 (65.4%)
S249C only	3 (11.5%)
R248C only	9 (34.6%)
Both S249C and R248C	5 (19.2%)

SD, standard deviation; FGFR3, fibroblast growth factor receptor 3.

**Table 2:** Paired urinary cytology and targeted urinary FGFR3 mutation results.

Urinary Cytology	FGFR3 Mutation Detected	No Targeted FGFR3 Mutation Detected	Total
Positive	5	3	8
Non-positive	12	6	18
Total	17	9	26

FGFR3-positive denotes detection of S249C, R248C, or both. Cytology-positive denotes SHGUC or HGUC; other cytology categories are grouped as non-positive for this descriptive analysis.

## Data Availability

The data presented in this study are available on request from the corresponding author due to privacy, legal and ethical restrictions.

## Abbreviations

The following abbreviations are used in this manuscript:

AKT	Protein kinase B
AS-PCR	Allele-specific polymerase chain reaction
AUC	Atypical urothelial cells
AUA	American Urological Association
BC	Bladder cancer
BCG	Bacillus Calmette–Guérin
BMI	Body mass index
CIS	Carcinoma in situ
CT	Computed tomography
ddPCR	Droplet digital polymerase chain reaction
DNA	Deoxyribonucleic acid
EAU	European Association of Urology
FAM	6-Carboxyfluorescein
FGFR	Fibroblast growth factor receptor
FGFR3	Fibroblast growth factor receptor 3
HGUC	High-grade urothelial carcinoma
LGUN	Low-grade urothelial neoplasm
MAPK	Mitogen-activated protein kinase
MIBC	Muscle-invasive bladder cancer
MPC	Protein precipitation reagent used in the MasterPure purification protocol
NCCN	National Comprehensive Cancer Network
NGS	Next-generation sequencing
NHGUC	Negative for high-grade urothelial carcinoma
NMIBC	Non-muscle-invasive bladder cancer
NTC	No-template control
PCR	Polymerase chain reaction
PI3K	Phosphoinositide 3-kinase
PLCγ	Phospholipase C gamma
PPV	Positive predictive value
NPV	Negative predictive value
RB1	Retinoblastoma 1 gene
RNA	Ribonucleic acid
SHGUC	Suspicious for high-grade urothelial carcinoma
STAT	Signal transducer and activator of transcription
SUO	Society of Urologic Oncology
TERT	Telomerase reverse transcriptase
TNM	Tumor–Node–Metastasis classification
TP53	Tumor protein p53 gene
TPS	The Paris System for Reporting Urinary Cytology
UC	Urothelial carcinoma
